# Tuo-Min-Ding-Chuan Decoction Alleviates Asthma via Spatial Regulation of Gut Microbiota and Treg Cell Promotion

**DOI:** 10.3390/ph18050646

**Published:** 2025-04-28

**Authors:** Yanfei Hong, Zheng Yang, Zirui Liu, Na Li, Jingbo Qin, Dongyu Ge, Guiying Peng, Ji Wang, Qi Wang

**Affiliations:** 1 Department of Immunology and Microbiology, School of Life Sciences, Beijing University of Chinese Medicine, Beijing 102401, China; yanfei_hong@bucm.edu.cn (Y.H.); nali@bucm.edu.cn (N.L.); 2 National Institute of TCM Constitution and Preventive Medicine, Beijing University of Chinese Medicine, Beijing 102401, China; yangzheng@bucm.edu.cn (Z.Y.); qinjb0315@163.com (J.Q.); wangqi710@126.com (Q.W.); 3 Wang Jing Hospital, China Academy of Chinese Medical Sciences, Beijing 100102, China; liuzirui19930518@163.com; 4 Scientific Research Center, School of Traditional Chinese Medicine, Beijing University of Chinese Medicine, Beijing 102401, China; gedongyu@sohu.com

**Keywords:** traditional Chinese medicine, probiotics, segmental gut microbiota distribution, regulatory T cells, allergic inflammation, microbiome–immune axis

## Abstract

**Objective**: Tuo-Min-Ding-Chuan decoction (TMDC), a traditional Chinese prescription, has demonstrated significant clinical efficacy in treating allergic asthma. This study aimed to investigate the mechanism of TMDC in treating asthma from the perspective of Treg cells and gut microbiota across distinct gut segments (jejunum, ileum, cecum, and colon). **Methods**: An ovalbumin (OVA)-induced asthma model was established in mice, followed by oral administration of TMDC at high, medium, and low dose. Immune cells and lung inflammation were examined to assess asthma severity. Microbial composition was determined by 16S rRNA sequencing. Antibiotic cocktail and *Lactobacillus rhamnosus* GG (LGG) were administrated to confirm the key role of specific bacteria. **Results**: TMDC attenuated lung inflammation (*p* < 0.01) and eosinophilic infiltration (*p* < 0.01) as well as IL-4 and IL-5 secretion (*p* < 0.01); it was also associated with an increase in Treg cells in the lung, small intestine (SI), and colon (*p* < 0.05). Meanwhile, TMDC restored the number of microbiota species and the Shannon index in the hindgut and reinstated beneficial bacteria, such as *Allobaculum* and *Turicibacter*, which were diminished in asthmatic mice. Notably, TMDC significantly enriched *Bifidobacterium* and *Lactobacillus*, particularly in the hindgut. *Lactobacillus* abundance was significantly correlated (*p* < 0.05) with Treg cells, IL-4, IL-5, and eosinophils. Furthermore, LGG supplementation restored elevated lung inflammation (*p* < 0.05) and decreased Treg cells (*p* < 0.01) due to antibiotic-induced microbiota depletion. **Conclusion**: TMDC alleviated asthma by promoting Treg cell expansion in a *Lactobacillus*-dependent manner across different gut segments, providing new insights into its therapeutic mechanisms.

## 1. Introduction

Allergic asthma is a chronic inflammatory disease characterized by airway hyperreactivity, shortness of breath, mucus hypersecretion, and inflammatory cell infiltration, significantly impacting patients’ quality of life [1,2,3]. The pathogenesis of asthma is closely related to the overactivation of the Th2 cell immune response, which leads to the production of type 2 cytokines such as IL-4 and IL-5 [4]. These cytokines promote eosinophil infiltration [5], thereby exacerbating airway inflammation. Despite the efficacy of inhaled corticosteroids and biologics targeting IL-4 or IL-5 in many asthma patients, a significant subset still experiences poor asthma control [6], highlighting the need for complementary therapeutic approaches. Regulatory T (Treg) cells maintain immune tolerance to allergens, limiting inflammation and promoting local tissue repair [7]. Recent research has underscored the substantial therapeutic potential of Tregs in allergic airway diseases [8].

The intestinal microbiota is integral to the host immune response, with dysbiosis implicated in asthma pathogenesis [9]. Compared to healthy controls, asthma patients exhibit elevated levels of *Clostridium* and *Eggerthella lenta* in their gut microbiota [10]. Additionally, reduced abundances of *Bifidobacterium* and *Faecalibacterium*, coupled with increased levels of *Candida* and *Rhodotorula*, correlate with a higher risk of allergies and asthma [11]. Specific strains of *Lactobacillus* bacteria (e.g., *Lactobacillus jensenii*) have been shown to modulate the immune system and reduce intergenerational transmission of allergic reactions, thereby lowering the risk of childhood asthma [12]. Clinical studies suggest that early supplementation with *Lactobacillus* can alter gut microbiota maturation in infants at high risk for asthma during their first year of life [13] and that *Lactobacillus* administration contributes to clinical improvement in children with asthma [14]. Recent research has demonstrated that *Lactobacillus delbrueckii* UFV-H2b20 augments lung Treg cell numbers and protects mice from experimental allergic asthma [15]. Similarly, oral administration of *Lactobacillus rhamnosus* increases Treg cell numbers and inhibits the allergic march [16].

Tuo-Min-Ding-Chuan decoction (TMDC) is a traditional Chinese medicine formula that has demonstrated significant clinical efficacy in treating allergic asthma. This formula, developed through years of clinical experience by Professor Wang Qi, a renowned expert in Chinese medicine, has shown excellent results in relieving clinical symptoms and reducing the frequency of asthma exacerbations [17,18]. However, the mechanisms underlying its therapeutic effects remain unclear. Previous studies have shown that TMDC effectively inhibits the infiltration of proinflammatory cells and mast cell degranulation, thereby attenuating airway hyperresponsiveness in asthmatic mice [17]. Additionally, our previous research found that TMDC treatment increased the abundances of *Bifidobacterium*, *Desulfovibrio*, and *Rikenella* in the feces of OVA-induced asthmatic mice [18]. These findings underscore the potential of TMDC to modulate the gut microbiota, which is increasingly recognized as a critical factor in asthma pathogenesis. Given the growing interest in the therapeutic potential of traditional Chinese medicine and the emerging insights into the gut–lung axis in asthma, probing the mechanisms of TMDC from microbial and immune standpoints presents both clinical relevance and scientific novelty.

Although the intestinal lumen is a continuous space, the gastrointestinal tract constitutes a heterogeneous ecosystem with diverse environments, leading to compartmentalized variations in microbial composition and functionality across different intestinal segments [19,20,21]. Feces, as the excretory residue of the digestive tract, share a similar community structure and function with the hindgut segment but cannot completely represent the entire intestinal microbiota. Our previous study focused on the fecal microbiota of asthmatic mice with or without TMDC administration. In the current study, we further investigated the effects of TMDC treatment on microbial alterations and Treg cells along distinct intestinal segments (e.g., jejunum, ileum, cecum, and colon) of OVA-induced asthmatic mice as well as the important role of gut microbiota in the treatment of asthma with TMDC. We aimed to identify key microbial species in different groups, with particular emphasis on the regional specificity of microbiota in relation to host health and disease, and to deepen the understanding of how TMDC modulates the gut microbiota.

## 2. Results

### 2.1. TMDC Alleviated the Lung Inflammation of Asthmatic Mice

**Figure 1:**
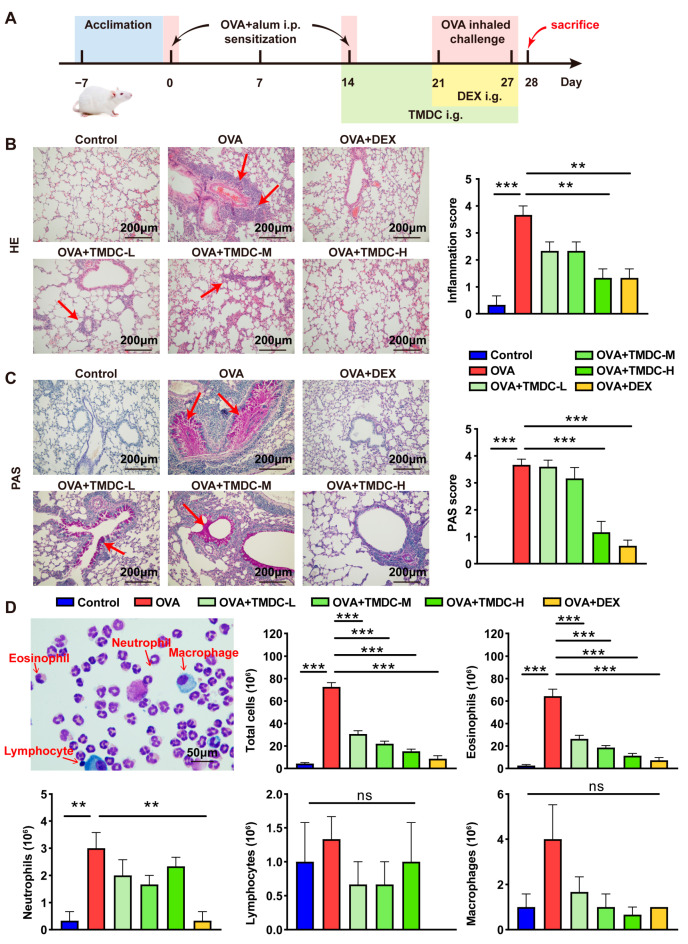
TMDC reduced lung inflammation in asthmatic mice. (**A**) Schematic illustration of OVA-induced asthmatic mice model; (**B**) typical pictures of HE staining of lung tissues (200×) and inflammation score, with arrows indicating inflammatory infiltration; (**C**) typical pictures of PAS staining of lung tissues (200×) and PAS score, with arrows indicating mucus secretion; (**D**) typical picture of BALF stained with Diff and total cell, eosinophil, neutrophil, lymphocyte, and macrophage count in BALF. ** *p* < 0.01; *** *p* < 0.001; ns: not significant.

To initially assess the therapeutic effect of TMDC on asthma, OVA-induced asthmatic mice were orally administered TMDC-H, TMDC-M, TMDC-L, or PBS, as depicted in Figure 1A. Lung tissues were subsequently collected and subjected to HE staining and PAS staining. HE staining revealed that asthmatic mice in the OVA group exhibited pronounced inflammatory cell infiltration around the trachea and thicker tracheal walls. In contrast, the bronchial wall was relatively thinner, and inflammatory infiltration was markedly diminished in the TMDC group and DEX group (Figure 1B). Moreover, PAS staining demonstrated an abundance of mucin-producing goblet cells lining the bronchial epithelium in OVA-challenged mice, which were significantly attenuated by TMDC and DEX (Figure 1C).

Next, we assessed the inflammatory cells in the BALF of mice using Diff-Quick staining. A representative image is presented in Figure 1D, with red arrows indicating immune cells. The total number of inflammatory cells, eosinophils, and neutrophils in the BALF of asthmatic mice was significantly higher compared to the control group. Notably, TMDC treatment significantly reduced this inflammatory infiltration, including both total inflammatory cells and eosinophils (Figure 1D).

These results demonstrate that TMDC alleviates lung inflammation by inhibiting the recruitment and infiltration of inflammatory cells, with the high dose exhibiting greater efficacy.

### 2.2. TMDC Reduced the Type 2 Cytokine by Promoting Treg Cells

The aforementioned results indicate that TMDC-H is more effective. Consequently, subsequent experiments were performed using the high dose to elucidate the underlying mechanisms of TMDC’s therapeutic effects.

**Figure 2:**
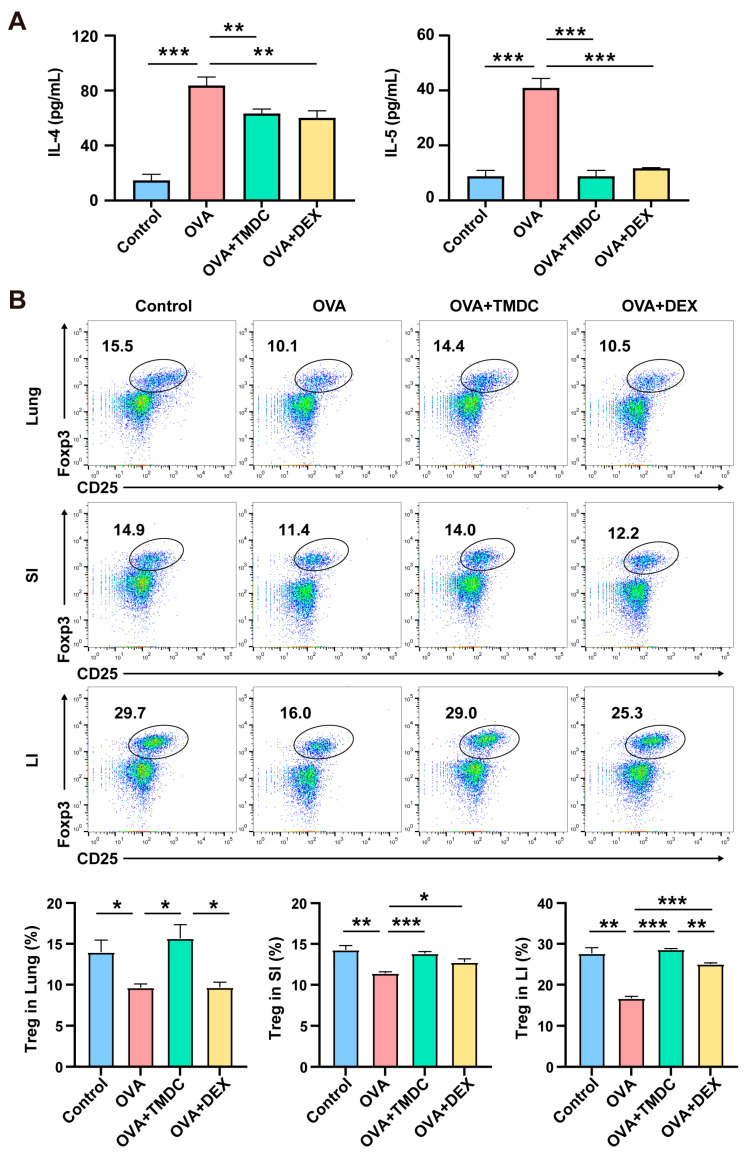
TMDC reduces type 2 cytokines by promoting Treg cells. (**A**) Levels of IL-4 and IL-5 in serum detected by ELISA; (**B**) Treg frequencies in lung, SI, and LI detected by flow cytometry; representative plots show the gates and frequencies of Treg cells. * *p* < 0.05; ** *p* < 0.01; *** *p* < 0.001.

Allergic asthma is typically characterized by high expression of IL-4, IL-5, and IL-13, along with elevated IgE levels and eosinophil infiltration; it is a prototypical type 2 inflammation-driven disease. Consistent with this, we found that the levels of IL-4 and IL-5 were significantly higher in the serum of the OVA group. In contrast, treatment with TMDC and DEX significantly inhibited the secretion of these cytokines (Figure 2A).

Treg cells are known to modulate Th2 responses and control allergic inflammation in asthma. We therefore assessed Treg cell frequency in lung, SI, and LI using flowcytometry. In contrast to the elevated type 2 cytokines, MXSG treatment significantly increased Treg cell frequency in these tissues (Figure 2B).

### 2.3. TMDC Improved the Microbial Composition Across Different Gut Segments

**Figure 3:**
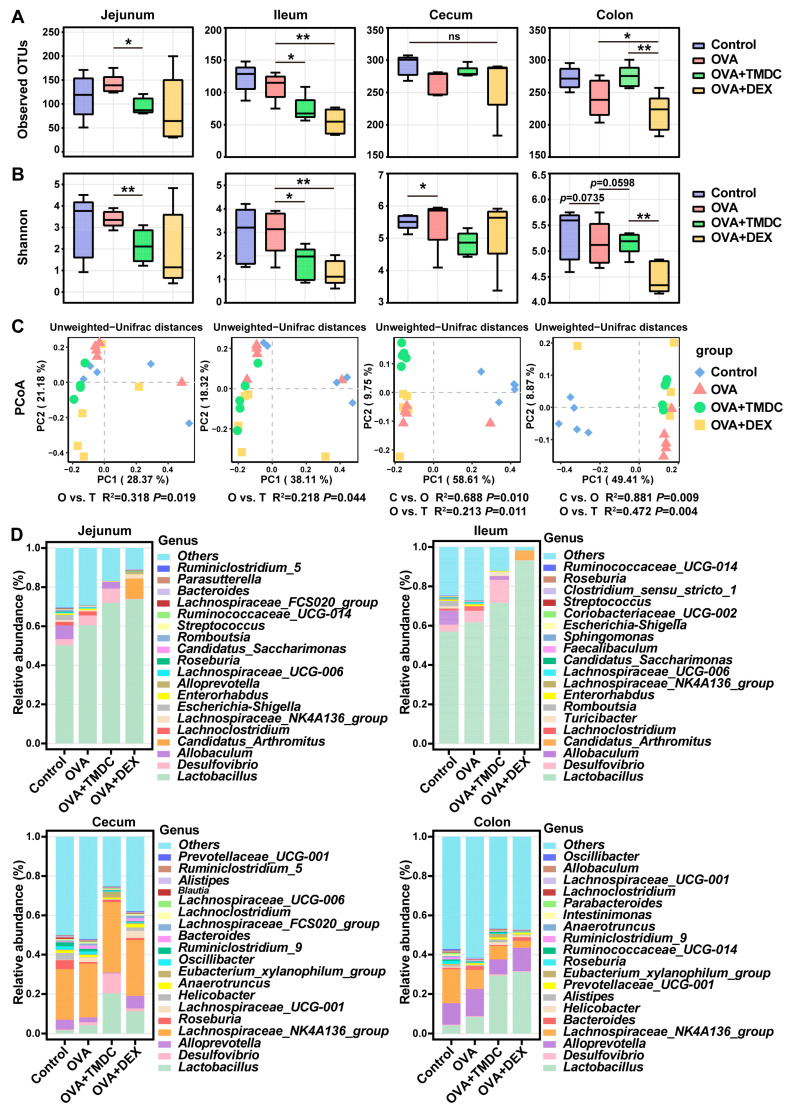
Effect of TMDC on microbial composition of different gut segments. (**A**) Observed OTUs; (**B**) Shannon index; (**C**) PCoA plots based upon Unweighted-Unifrac distances with PERMANOVA analysis; (**D**) predominant genera at different gut segments. * *p* < 0.05; ** *p* < 0.01; ns: not significant.

Previous research has indicated that TMDC can alleviate asthma by modulating the fecal bacterial community. To further investigate the effects of TMDC across different gut segments, we conducted 16S rRNA gene sequencing on samples from the jejunum, ileum, cecum, and colon. Phylogenetic trees illustrated the evolutionary relationships between various microbial species across these segments (Supplementary Figure S1). Community clustering heatmaps highlighted differences in the composition and abundance of microbial communities across samples and gut segments (Supplementary Figure S2). Alpha diversity metrics, including observed OTUs and Shannon index, were significantly higher in the cecum and colon compared to the jejunum and ileum across all groups (Supplementary Figure S3), aligning with prior findings [21]. These results underscore the importance of characterizing the microbial changes in distinct gut regions. Moreover, TMDC notably reduced observed OTUs in the jejunum and ileum but increased them in the colon, indicating enhanced microbial richness in the hindgut (Figure 3A). Additionally, TMDC led to a decrease in the Shannon index across all intestinal segments, suggesting a reduction in microbial community diversity (Figure 3B). This outcome may be due to an increase in the relative abundance of specific taxa within the community, resulting in a less even distribution of species. PCoA and PERMONOVA analysis further demonstrated significant separation of overall community structures in TMDC-treated mice compared to asthmatic mice across all gut segments (Figure 3C). These findings indicate that TMDC can remodel the microbiota structure along the entire intestinal tract of asthmatic mice.

Consistent with these observations, community composition analysis revealed notable differences in microbiota compositions among different groups (Figure 3D, Supplementary Figures S4 and S5). TMDC increased the relative abundance of Firmicutes and Proteobacteria while decreasing that of Bacteroidetes, which are the three dominant phyla across the gut segments (Supplementary Figures S4 and S5). Notably, mice in the OVA+TMDC group exhibited higher proportions of the genus *Lactobacillus,* particularly in the small intestine and colon (Figure 3D).

### 2.4. Specific Microbes Affected by TMDC Across Different Gut Segments

**Figure 4:**
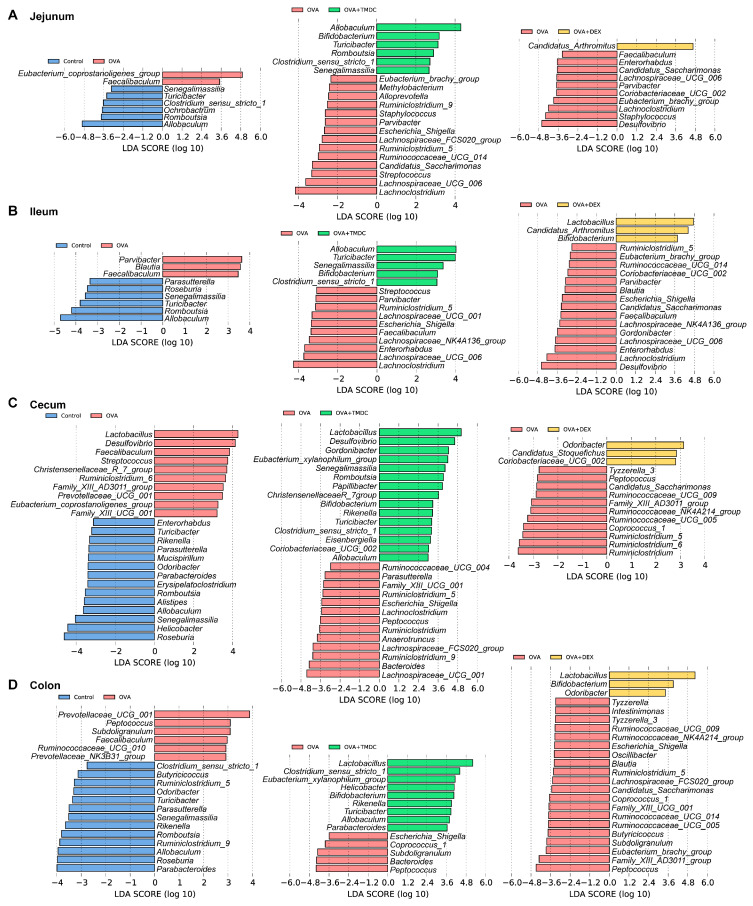
Specific microbial changes in different gut segments affected by TMDC. Differentially abundant genera in (**A**) jejunum, (**B**) ileum, (**C**) cecum, and (**D**) colon between different two groups generated from LEfSe with an LDA score (threshold ≥ 2).

Subsequently, to identify bacterial indicators among different groups, LEfSe analysis was performed based on the top 200 genera (Figure 4). As shown in Figure 4, in the jejunum of asthmatic mice compared with control mice, 2 genera had higher abundance; in the ileum, 3 genera; in the cecum, 10 genera; and in the colon, 6 genera. After TMDC treatment, 6 genera were enriched in jejunal samples, 5 in ileal samples, 15 in cecal samples, and 9 in colonial samples. In contrast, the effect of DEX on gut bacteria appeared to be weaker, with four genera enriched in the SI and six genera in the LI.

### 2.5. Lactobacillus Exhibited Significant Correlations to Inflammatory Cells and Cytokines

**Figure 5:**
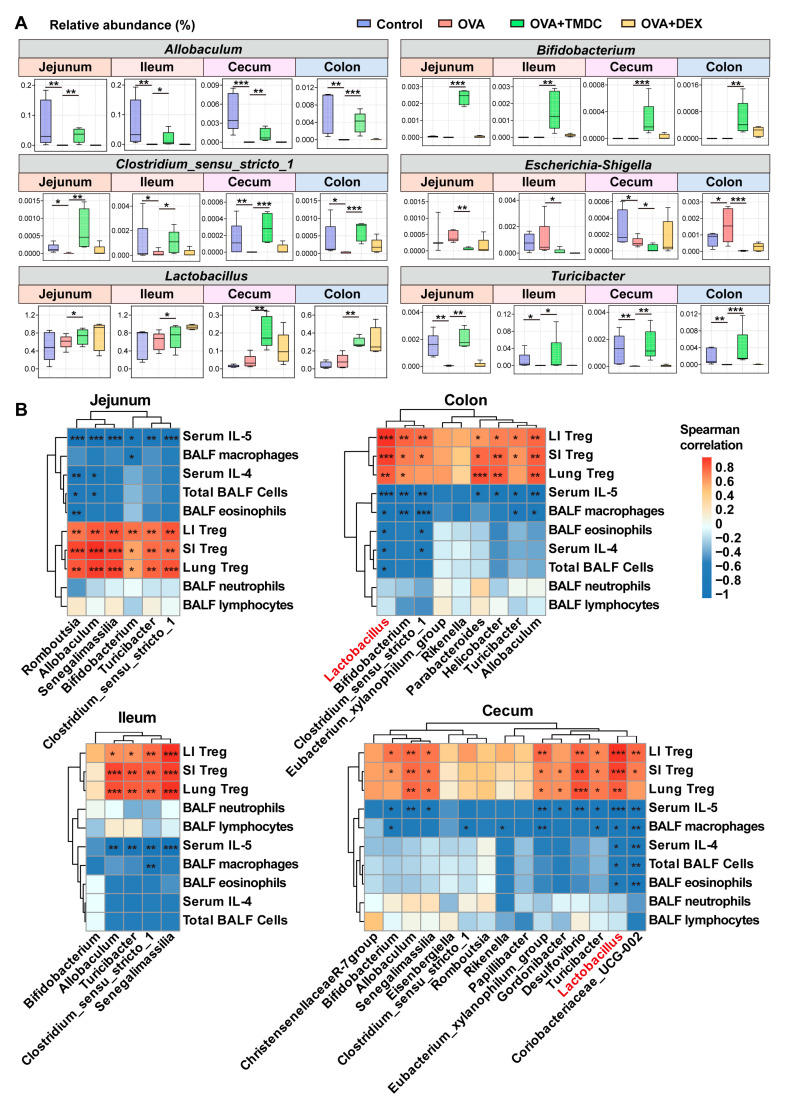
TMDC-associated specific microbes exhibit significant correlations to Treg and cytokines. (**A**) Targeted genera relative abundance in jejunum, ileum, cecum, and colon among different groups; (**B**) correlation between specific microbes and immune indicators among different gut segments. In the correlation analysis, the red color highlights a specific microbe of interest, which shows a high number of significant correlations and strong correlation coefficients. * *p* < 0.05; ** *p* < 0.01; *** *p* < 0.001.

Interestingly, the genera *Allobaculum*, *Clostridium_sensu_stricto_1*, and *Turicibacter*, which were less abundant in asthmatic mice compared to controls, were dramatically enriched by TMDC. The potential pathogenic bacterium *Escherichia-Shigella*, which had a higher abundance in the SI and colon of asthmatic mice, was notably suppressed by TMDC (Figure 5A). Meanwhile, TMDC strikingly elevated the abundance of *Bifidobacterium*, a well-known probiotic (Figure 5A). Additionally, the genus *Lactobacillus*, which was less abundant in the jejunum and ileum of asthmatic mice, was restored by TMDC and significantly enriched in the hindgut (Figure 5A).

Further correlation analyses revealed that *Allobaculum*, *Clostridium_sensu_stricto_1*, and *Turicibacter* were negatively correlated to serum IL-5 and positively associated with Treg cells in the lung, small intestine, and large intestine of asthmatic mice (Figure 5B). Conversely, *Lactobacillus* in the hindgut showed negative correlations with BALF inflammatory cells as well as serum IL-4 and IL-5 and positive associations with Treg cells in the lung, SI, and LI (Figure 5B). These results suggest that the alterations of the inflammatory status in the lungs of asthmatic mice by TMDC might be partly mediated by *Lactobacillus*.

### 2.6. Lactobacillus Supplementation Restores Antibiotic-Induced Diminished Therapeutic Effect of TMDC

**Figure 6:**
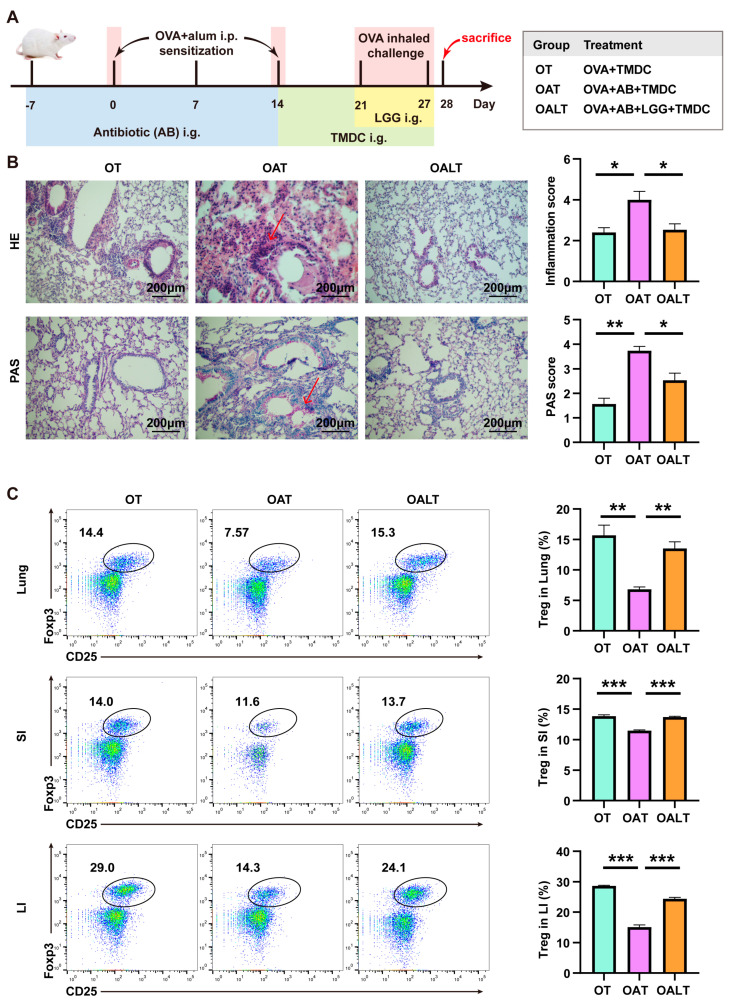
*Lactobacillus* supplementation restores the diminished effect of antibiotic-induced TMDC therapy. (**A**) Schematic diagram of a mouse model; (**B**) typical images of HE and PAS staining of lung tissues (200×), with arrows indicating inflammatory infiltration and mucus secretion; (**C**) Treg frequencies in lung, SI, and LI detected by flow cytometry; representative plots show the gates and frequencies of Treg cells. * *p* < 0.05; ** *p* < 0.01; *** *p* < 0.001.

To further validate the crucial role of *Lactobacillus* in the therapeutic effects of TMDC on asthma, asthmatic mice were administered antibiotics via gavage to deplete intestinal bacteria and were subsequently treated with *Lactobacillus rhamnosus* GG (LGG) orally (Figure 6A). Histological examination of lung tissue via HE and PAS staining revealed that mice in the OAT group exhibited increased inflammatory cell infiltration around the trachea and elevated mucin level, indicating that antibiotic-induced gut bacteria depletion diminished the therapeutic efficacy of TMDC. In contrast, the therapeutic effects of TMDC were restored following LGG replenishment, as evidenced by the reduced inflammatory infiltration and mucin levels in the OALT group (Figure 6B). Additionally, Treg cell frequences in the lung, SI, and LI were decreased following gut microbiota clearance but were restored after LGG replenishment (Figure 6C). These findings suggest that Treg cells and pulmonary inflammation were highly correlated with intestinal microbes, particularly *Lactobacillus*, and that TMDC alleviates OVA-induced asthmatic inflammation in a microbiota-dependent manner.

## 3. Discussion

TMDC is a traditional Chinese prescription designed to treat allergic asthma, guided by the theory of constitution. It has demonstrated significant clinical efficacy in reducing the frequency of asthma attacks [17]. To further explore its therapeutic potential, we investigated the prophylactic effects of TMDC in a murine model of asthma. Our results showed that TMDC alleviated the inflammatory phenotype of the lung and significantly reduced eosinophil accumulation in the BALF of asthmatic mice. Despite these promising findings, the exact mechanisms underlying TMDC’s therapeutic effects remain unclear. In our previous study, we analyzed TMDC using liquid chromatography–mass spectrometry (LC-MS) and identified 223 substances by comparing them with a database. These substances include phenylpropanoids, flavonoids, flavonoid glycosides, cyanogenic glycosides, triterpenoid saponins, triterpenoids, amino acids, and organic acids. Most of these compounds are known for their anti-inflammatory, anti-allergic, and anti-asthmatic properties. Ours study also demonstrated that TMDC reduced airway hyperresponsiveness (AHR) and decreased pulmonary eosinophil numbers and total IgE levels in asthmatic mice [17]. In vitro experiments showed that TMDC significantly suppressed mast cell degranulation [17], suggesting that it may reduce the allergic response and prevent asthma by inhibiting mast cell degranulation and suppressing eosinophil numbers. As a multi-targeted therapy, traditional Chinese medicine like TMDC holds great promise in regulating immune homeostasis. This integrative approach offers new therapeutic strategies for managing allergic asthma.

Treg cells represent the guardians of the immune homeostasis, capable of restraining all major types of inflammatory responses. Treg cells produce immunomodulatory cytokines such as IL-10 and TGF-β in a Foxp3-dependent manner. They also convert extracellular ATP, a potent pro-inflammatory mediator, into its anti-inflammatory product, adenosine, thereby controlling persistent inflammation [22]. Lung-targeting CAR Treg cells have demonstrated favorable amelioration against the main features of experimental airway inflammation [23]. In asthma patients, upregulation of Notch4, Wnt, and Hippo in circulating Treg cells subvert them into Th2 and Th17 cells, diminishing Treg cell-mediated suppression and contributing to increase disease severity [24]. Pi-Pa-Run-Fei-Tang (PPRFT), an empirical TCM prescription for treating asthma, contains radix *Glycyrrhizae*, which restores the Th17/Treg balance to alleviate OVA-induced asthma [25]. Additionally, Ephedrae Herba polysaccharides (PE), an ingredient in TMDC, inhibit the inflammation in asthmatic rats by regulating Th1/Th2 and Th17/Treg cell immune imbalances [26].

The gut microbiota plays a crucial role in regulating host immunity, with the gut–lung axis garnering significant attention in recent years. Several studies have demonstrated that traditional Chinese medicine exerts its therapeutic effects through this axis. For instance, Gegen Qinlian decoction alleviates lung inflammation in experimental colitis by inhibiting the recruitment of inflammatory myeloid cells and restoring microbial homeostasis [27]. Dachengqi decoction relieves asthma by reducing group 2 innate lymphocytes in a microbiota-dependent manner [28]. Xuanbai Chengqi decoction ameliorates COPD through gut microbiota remodeling and correction of the Th17/Treg imbalance [29]. Our prior research indicated that TMDC can modulate the fecal microbiota of asthmatic mice by enriching specific taxa, including *Rikenellaceae_RC9_gut_group*, *Bifidobacterium*, *Rikenella*, *Butyricimonas*, *Desulfovibrio*, and *Prevotella* [18]. However, the functional heterogeneity of individual intestinal segments leads to regional variations in microbial populations [19,20,21]. Given that the feces, which share a similar community structure and function with the hindgut, do not fully represent the entire intestinal microbiota, we further investigated the microbial changes in the jejunum, ileum, cecum, and colon of asthmatic mice treated with TMDC. Our results revealed significant separation of gut microbiota across the four intestinal segments. In the small intestine, Firmicutes and Proteobacteria were predominant, while Bacteroidetes increased and Firmicutes and Proteobacteria decreased from the small to the large intestine, aligning with previous studies [30]. At the genus level, *Lactobacillus* was dominant in the jejunum and ileum but decreased significantly in the large intestine. In contrast, several genera were more abundant in the cecum and colon compared to the jejunum and ileum, including *Alloprevotella*, *Prevotellaceae_UCG-001*, *Lachnospiraceae_NK4A136_group*, *Lachnospiraceae_UCG-001*, *Lachnospiraceae_FCS020_group*, and *Lachnospiraceae_UCG-006*. Again, these findings underscore the spatial preferences of microbiota for colonizing different gut regions.

Throughout the intestinal tract, TMDC administration resulted in a reduction in microbial population diversity, likely attributable to a substantial increase in the abundance of *Lactobacillus,* particularly in hindgut. Correlation analyses indicated that *Lactobacillus* plays a crucial role in the therapeutic effects of TMDC on asthma. This association was further confirmed using antibiotic-treated and LGG-supplemented asthmatic mice. The therapeutic efficacy of TMDC was compromised following the clearance of gut microbes and was restored upon LGG supplementation, highlighting the close correlation between the therapeutic effects of herbal medicine and the gut microbiota. Research has suggested that the gut microbiota can enhance the bioavailability of herbal medicine components through metabolic processes [31]. A systematic review and meta-analysis revealed that probiotics significantly ameliorated lung inflammation and symptom severity and reduced the frequency of acute asthma exacerbations in patients with asthma, although they had no significant impact on lung function (e.g., FEV1) [32]. While some clinical studies have shown that LGG does not directly prevent or treat asthma [33,34], LGG exhibited a favorable anti-inflammatory effect in a mouse model of asthma [35,36,37]. Moreover, a combination therapy comprising turmeric powder (TP) and LGG (synbiotic) was found to be more effective in mitigating allergic inflammation, such as by increasing Treg cells, compared to individual treatments. LGG can promote Treg cell function by augmenting butyrate production [38]. Consistent with these findings, the present study demonstrated that TMDC can treat asthma by boosting Treg cells in the presence of the probiotic LGG, thereby further validating the microbiota-dependent efficacy of TMDC in treating allergic asthma.

*Lactobacillus*, a quintessential probiotic, exerts immunomodulatory effects on the host through multiple pathways. It regulates the function of Treg cells by stimulating the production of short-chain fatty acid (SCFA), particularly butyrate and propionate [39,40]. SCFAs bind to the free fatty acid receptor 2 (FFAR2, also known as GPR43) [41], thereby modulating the quantity and activity of colonic Treg cells [42]. Moreover, SCFAs promote Treg cell development and IL-10 secretion by inhibiting histone deacetylase (HDAC) [43]. This mechanism may elucidate how TMDC enhances Treg cell regulation via *Lactobacillus*.

In this study, we elucidated the efficacy of TMDC using an asthma mouse model and analyzed its effects on microbial communities across different intestinal segments via 16S rRNA sequencing. We further validated the pivotal role of specific microbiota through antibiotic and probiotic interventions. However, our study still has some limitations. While we confirmed a strong association between TMDC treatment and *Lactobacillus* through antibiotic-mediated bacteria clearance, the precise role of *Lactobacillus* in TMDC’s therapeutic effects on asthma remains to be determined. Additionally, 16S rRNA sequencing offers limited accuracy for species identification and functional insights. Future research should employ metagenomic sequencing to more comprehensively elucidate the composition and function of microbial communities as well as the interactions between bacterial functions, metabolites, and immune cells. This would provide deeper mechanistic insights into the treatment of asthma with TMDC. Future research should also address the lack of longitudinal microbiota tracking and human clinical validation.

## 4. Materials and Methods

### 4.1. Animals

Female BALB/c mice, aged 6–8 weeks, were purchased from the Beijing Vital River Laboratory Animal Technologies Co. Ltd (Beijing, China). and housed in the specific pathogen-free facility at the Animal Center of Beijing University of Chinese Medicine. The animals were acclimatized for 7 days prior to the experiment, maintained at 20–23 °C under a 12 h/12 h light-dark cycle, and provided with ad libitum access to water and food. This study was approved by the Experimental Animal Ethics Committee of Beijing University of Chinese Medicine (BUCM-4-2021111301-4168), the approval date was 13 November 2021, and the experimental process was in accordance with the guidelines related to animal ethics.

### 4.2. Preparation of TMDC Decoction

TMDC is composed of 12 botanical drugs, with the exact composition detailed in Table 1. The botanical drugs were first soaked in distilled water for 30 min at room temperature (approximately 25 °C). Subsequently, the mixture was brought to a boil over high heat, reaching a temperature of 100 °C, and maintained at this temperature for 10 min. The mixture was then simmered at a reduced temperature of 90 °C for an additional 20 min. This extraction process was repeated twice to ensure thorough extraction of active components. The decoctions obtained from the two rounds of boiling were combined and filtered through a double layer of gauze to remove any residual plant material. The filtered decoction was transferred to a water bath maintained at 80 °C, where it was heated to facilitate evaporation and concentration. The concentrated decoction was stored at −20 °C to maintain stability and preserve the active components. Prior to use, the decoction was thawed at room temperature and diluted to the desired concentration for gavage administration. The dose of oral administration is 0.2 mL per mouse. The middle dose of TMDC (TMDC-M) was determined by calculating the clinical equivalent dose using a conversion coefficient from human to mouse based on body surface area. The low- and high-dose of TMDC (TMDC-L and TMDC-H) were 1/2 or 2 times of the medium concentration, respectively. The detailed calculation formula based on the standard body weight of adults and mice is as follows:

Clinical equivalent dose of one mouse (TMDC-M, g/mL)
= (clinical dose × conversion coefficient)/gavage volume
= (156 g × 0.0026)/0.2 mL = 2.028 g/mL

### 4.3. Establishment of OVA-Induced Asthmatic Mouse Model

The OVA-induced asthma model is a well-established paradigm for allergic asthma, effectively replicating the hallmark features of human asthma. Its high reproducibility and robustness render it a preferred choice for screening and evaluating the efficacy of anti-asthma therapeutic agents. The mouse asthma model was established using ovalbumin as previously described [17]. Briefly, mice were randomly assigned to six groups (n = 6 per group): control, OVA, OVA+TMDC-L, OVA+TMDC-M, OVA+TMDC-H, and OVA+ dexamethasone (OVA+DEX). Mice in the OVA group were sensitized intraperitoneally on days 0 and 14 with 20 μg OVA (Sigma, St. Louis, MO, USA) emulsified in PBS and Imject™ Alum (Thermo Fisher Scientific, Waltham, MA, USA) at a 1:3 ratio, with constant mixing to a total volume of 200 μL. From days 21 to 27, these mice were challenged with aerosolized 1% OVA for 30 min daily, while mice in the control group received an equivalent volume of phosphate-buffered saline (PBS). TMDC-treatment groups received the corresponding doses of TMDC orally once daily from day 14 to 27, 30 min prior to aerosolization. The OVA+DEX group received dexamethasone (Sigma, 1 mg/kg/d) from days 21 to 27, while the control group received PBS. All mice were sacrificed on day 28.

### 4.4. Antibiotic Treatment and LGG Treatment in an Asthmatic Mouse Model

Mice were randomly divided into three groups (three mice per group): OT, OAT, and OALT. The murine asthma model was established with ovalbumin as described above. All mice were orally administrated with the high dose of TMDC. Mice in the OAT group and OALT group were administrated with an antibiotic (AB) cocktail containing ampicillin, vancomycin, metronidazole, neomycin, and amphotericin-B. Amphotericin-B (1 mg/kg, INALCO, Milan, Italy) was administered by gavage every 12 h for the first 7 days. Then, the antibiotic cocktail containing amphotericin-B (1 mg/kg, INALCO, USA), vancomycin (50 mg/kg, INALCO, USA), neomycin (100 mg/kg, INALCO, USA), and metronidazole (100 mg/kg, INALCO, USA) was administered by gavage every 12 h for the next 14 days. Ampicillin (1 g/L, INALCO, USA) was added ad libitum to the drinking water at 7-day intervals throughout the 21-day period. Mice in OALT group were administered with *Lactobacillus rhamnosus* GG (LGG, 5 × 10^7^ CFU/mL) by gavage from days 21 to 27. All mice were sacrificed on day 28.

### 4.5. Collection of Bronchoalveolar Lavage Fluid (BALF) for Diff-Quick Staining

Mice were sacrificed 24 h after the last challenge. A catheter was inserted into the trachea, and BALF was collected by gently flushing the lung with 0.8 mL PBS three times through the trachea. After the BALF was centrifuged at 2000 r/min for 5 min, the supernatant was collected, and the remaining cells were resuspended with 1 mL of PBS containing 5% BSA. BALF cells were spun onto microscope slides by CytoFuge and were used to identify different inflammatory cells by Diff-Quick Staining (Solarbio, Beijing, China). Differential cell counts were performed by counting 400 cells per slide using a high-magnification microscope.

### 4.6. Tissue Histology and Analysis

Lung tissues were collected and fixed in 4% phosphate-buffered formalin and embedded in paraffin for sectioning. Sections (5 mm) were used for hematoxylin and eosin (HE) staining and periodic acid-Schiff (PAS) staining. These tissues were examined under a light microscope, and their pathology was scored in a blinded way according to the method described by Dong et al. [44].

### 4.7. Detection of Cytokine Levels by ELISA

The mouse blood was collected and centrifuged at 3000 rpm for 10 min to obtain serum samples for the following ELISA test. The serum was processed for the measurement of cytokines, including IL-4 and IL-5, by ELISA (BioLegend, San Diego, CA, USA) according to the manufacturer’s instructions.

### 4.8. Preparation of Single-Cell Suspensions of Lung, SI, and Colon

Fresh lung, small intestine, and colon tissues were collected and rinsed in PBS. For single-cell extraction from intestinal tissues, the tissues were first dissected longitudinally and swabbed with PBS to remove intestinal contents. They were then cut into 1–2 cm long pieces and incubated with 20 mL of D-Hanks solution containing 5 mM EDTA and 1 mM DTT in a shaker at 37 °C for 20 min, repeated twice to remove epithelial cells. Subsequently, the tissues were cut into smaller pieces (approximately 3–5 mm square) and incubated with 5 mL RPMI-1640 containing 2 mg/mL collagenase type III (Worthington, CLS-3, enzyme activity ≥100 units/mg dry weight) and 50 μg/mL DNase I (Roche, Basel, Switzerland) for 30 min. Lung tissues were minced and digested with 5 mL RPMI-1640 containing 2 mg/mL collagenase type IV (Worthington, Lakewood, NJ, USA) and 50 μg/mL DNase I (Roche, Switzerland, enzyme activity ≥2000 U/mg) at 37 °C on a shaking bed for 30 min.

For single-cell extraction from lung tissues, the lung tissues were first chopped after being immersed in PBS. They were then digested with 5 mL of RPMI-1640 containing 2 mg/mL collagenase type IV (Worthington, CLS-4, enzyme activity ≥160 units/mg dry weight) and 50 μg/mL DNase I (Roche, Switzerland, enzyme activity ≥2000 U/mg) at 37 °C for 30 min on a shaker.

All digested tissues fluids were then filtered through a 70 μm cell strainer, and lymphocytes were isolated by 40% isotonic Percoll density gradient separation after erythrocyte lysis. Single-cell suspensions from all tissues were prepared for subsequent flow cytometry staining.

### 4.9. Analysis of Treg Cells by Flow Cytometry

Single-cell suspensions were preincubated with purified anti-mouse CD16/32 at 4 °C for 10 min to block the nonspecific binding to Fc receptors before staining. Then, the cells were stained with 100 mL FACS containing 0.5 μL fluorochrome-conjugated antibodies for surface staining: APC-Cy7 anti-mouse CD3, FITC anti-mouse CD4, and PerCP-Cy5.5 anti-mouse CD25. After fixation and permeabilization using the kit, cells were intracellular stained with APC anti-mouse Foxp3. Stained cells were detected using Cytoflex flow cytometer (Beckman Coulter, Brea, CA, USA) and analyzed with FlowJo V10 software.

### 4.10. 16S rRNA Gene Sequencing and Raw Data Analysis

Total genomic DNA was obtained from jejunal, ileal, cecal, and colonic digesta using the QIAamp DNA Stool Mini Kit (Qiagen Ltd., Hilden, Germany) according to the manufacturer’s guidelines. The V3–V4 hypervariable region of the bacterial 16S rRNA gene was amplified using the universal primer pair 338F (ACTCCTACGGGAGGCAGCAG) and 806R (GGACTACHVGGGTWTCTAAT). Then, the PCR products were purified, quantified, pooled into equivalent quantities, and finally processed with the Illumina MiSeq platform for creating paired-end raw reads with 300bp in length.

Raw FASTQ files were de-multiplexed and quality-filtered using QIIME (version 1.9). In brief, low-quality sequences with a length of <220 bp or >500 bp, with an average quality score of <20, and containing >3 uncertain bases were removed. The remaining high-quality reads were then clustered into operational taxonomic units (OTUs) at a 97% similarity cutoff using UPARSE (version 2.7.1), and chimeric sequences were removed using UCHIME. Taxonomy assignment of OTUs at each level (phylum, class, order, family, genus, and species) was conducted using the RDP classifier against the SILVA 16S rRNA gene database (Release 132) with a confidence threshold of 0.70.

Observed OTUs and Shannon indexes were calculated by QIIME (version 1.9) to estimate alpha diversity. Bar plots were visualized to present the bacterial community composition using the “vegan” package in R (version 3.6.0). Principal coordinates analysis (PCoA) was performed based on Bray–Curtis and Jaccard distances using QIIME (version 1.9) to evaluate beta diversities. In addition, permutational multivariate analysis of variance (PERMANOVA, with 1000 Monte Carlo permutations) was carried out based on distance matrices to compare the difference of community structures between groups using the Adonis function available in the “vegan” package of R (version 3.6.0). The differentially abundant bacteria taxa among groups were identified using discriminant analysis (LDA) effect size (LEfSe) analysis. Only taxa with an average relative abundance greater than 0.01% were included.

### 4.11. Statistical Analysis

SPSS software (Version 22.0, USA) was used for data analysis. Two groups were compared using a Student’s *t*-test. Statistical significance from more than two groups was calculated by one-way ANOVA. The Kruskal–Wallis test was used to estimate non-normally distributed data. *p*-values for multiple comparisons were corrected using a false-discovery rate (FDR) as described by Benjamini and Hochberg [45]. Correlations between different bacteria taxa and inflammatory indicators were evaluated using Spearman’s correlation analysis with the “heatmap” package in R (version 3.3.1). *p*-value ≤ 0.05 was considered to be statistically significant. Data are presented as means ± SEM.

## 5. Conclusions

**Figure 7:**
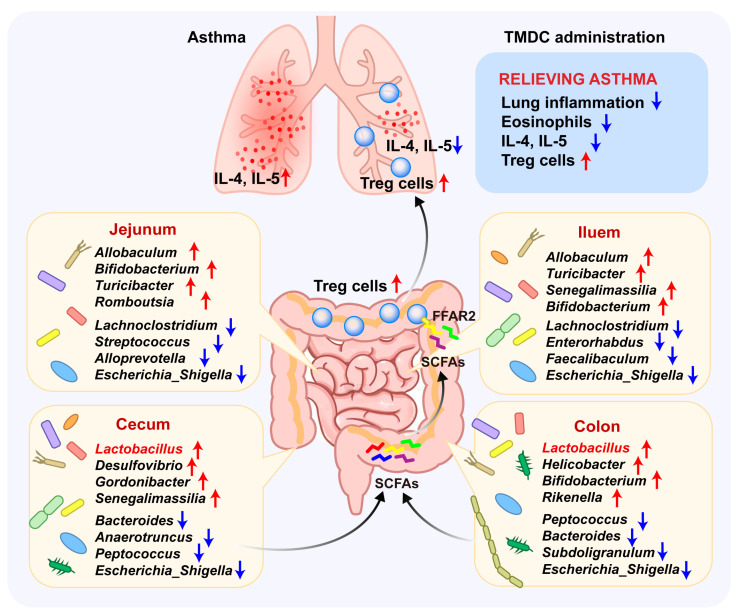
Integrative diagram showing the main results obtained from the present work: TMDC alleviates asthma by promoting Treg cell counts through modulation of microbial communities across different intestinal segments, particularly by increasing Lactobacillus in the hindgut, potentially via the short-chain fatty acid pathway.

In conclusion, our study demonstrates that TMDC attenuates asthma by promoting Treg cells, inhibiting eosinophil infiltration, and reducing type 2 cytokines—effects that are associated with the modulation of microbiota distribution across different intestinal segments (Figure 7). Notably, beneficial bacteria such as *Allobaculum* and *Turicibacter*, which are diminished in the intestines of asthmatic mice, are significantly restored by TMDC, leading to an increase in *Bifidobacterium* and *Lactobacillus* populations, especially in the hindgut. Further experiments using antibiotics to deplete the microbiota and subsequent supplementation with LGG confirmed the critical role of *Lactobacillus* in mediating TMDC’s therapeutic effects. Our findings offer novel insights into the biological mechanisms underlying asthma treatment and desensitization, highlighting the importance of the spatial distribution of the gut microbiota and its potential regulatory role in asthma. Future research should focus on metagenomic or metabolomic analyses of the TMDC-treated microbiota and validation studies in human cohorts to further elucidate these mechanisms.

## Figures and Tables

**Table 1:** Components of TMDC Prescription.

Component	Chinese Name	Amount (g)
*Gypsum fibrosum [Sulfates]*	Shi Gao	30
*Prunus mume (Siebold) Siebold & Zucc [Rosaceae]*	Wu Mei	20
*Fagopyrum cymosum (Trevir.) Meisn. [Polygonaceae]*	Jin Qiao Mai	15
*Reynoutria multiflora (Thunb.) Moldenke [Polygonaceae]*	Shou Wu Teng	15
*Ganoderma lucidum (Leyss. ex Fr.) Karst. [Polyporaceae]*	Ling Zhi	10
*Saposhnikovia divaricata (Turcz. ex Ledeb.) Schischk [Apiaceae]*	Fang Feng	10
*Gastrodia elata Blume [Orchidaceae]*	Tian Ma	10
*Ephedra sinica Stapf [Ephedraceae]*	Ma Huang	10
*Prunus armeniaca L. [Rosaceae]*	Xing Ren	10
*Cicadae periostracum [Cicadidae]*	Chan Tui	10
*Bombyx batryticatus [Silkworm pilgrimaging]*	Jiang Can	10
*Glycyrrhiza glabra L. [Fabaceae]*	Sheng Gan Cao	6

## Data Availability

The raw data supporting the conclusions of this article will be made available by the authors on request.

## Supplementary Materials

The following supporting information can be downloaded at https://www.mdpi.com/article/10.3390/ph18050646/s1. Figure S1. Phylogenetic trees of gut microbiota across different intestinal segments. (A) Jejunum, (B) Ileum, (C) Cecum, (D) Colon. Figure S2. Community clustering analyses of gut microbiota across different intestinal segments. (A) Jejunum, (B) Ileum, (C) Cecum, (D) Colon. Figure S3. The LI exhibited a higher alpha diversity than the SI of all mice. The alterations in the different intestinal microbiota structure of asthmatic mice. (A) Observed OTUs; (B) Shannon index. ** *p* < 0.01, *** *p* < 0.001. Figure S4. Abundant phyla at different gut segments of mice. Figure S5. Differentially abundant phyla at different gut segments among different groups.

## Abbreviations

The following abbreviations are used in this manuscript:

AHR	airway hyperresponsiveness
BALF	bronchoalveolar lavage fluid
DEX	dexamethasone
H&E	hematoxylin and eosin
LC-MS	liquid chromatography–mass spectrometry
LI	large intestine
OTU	operational taxonomic unit
OVA	ovalbumin
PAS	periodic acid-Schiff
PBS	phosphate-buffered saline
PTS	phosphotransferase system
SI	small intestine
TCM	traditional Chinese medicine
TMDC	Tuo-Min-Ding-Chuan decoction
