# Supplementary material for: Tuo-Min-Ding-Chuan Decoction Alleviates Asthma via Spatial Regulation of Gut Microbiota and Treg Cell Promotion
Source: Pharmaceuticals (Basel). 2025 Apr 28;18(5):646. doi: 10.3390/ph18050646 (PMC12115061; doi:10.3390/ph18050646)
Supplement: Supplementary file 1 [file pharmaceuticals-18-00646-s001.zip › pharmaceuticals-3577541-supplementary.pdf]

Supplementary Figures

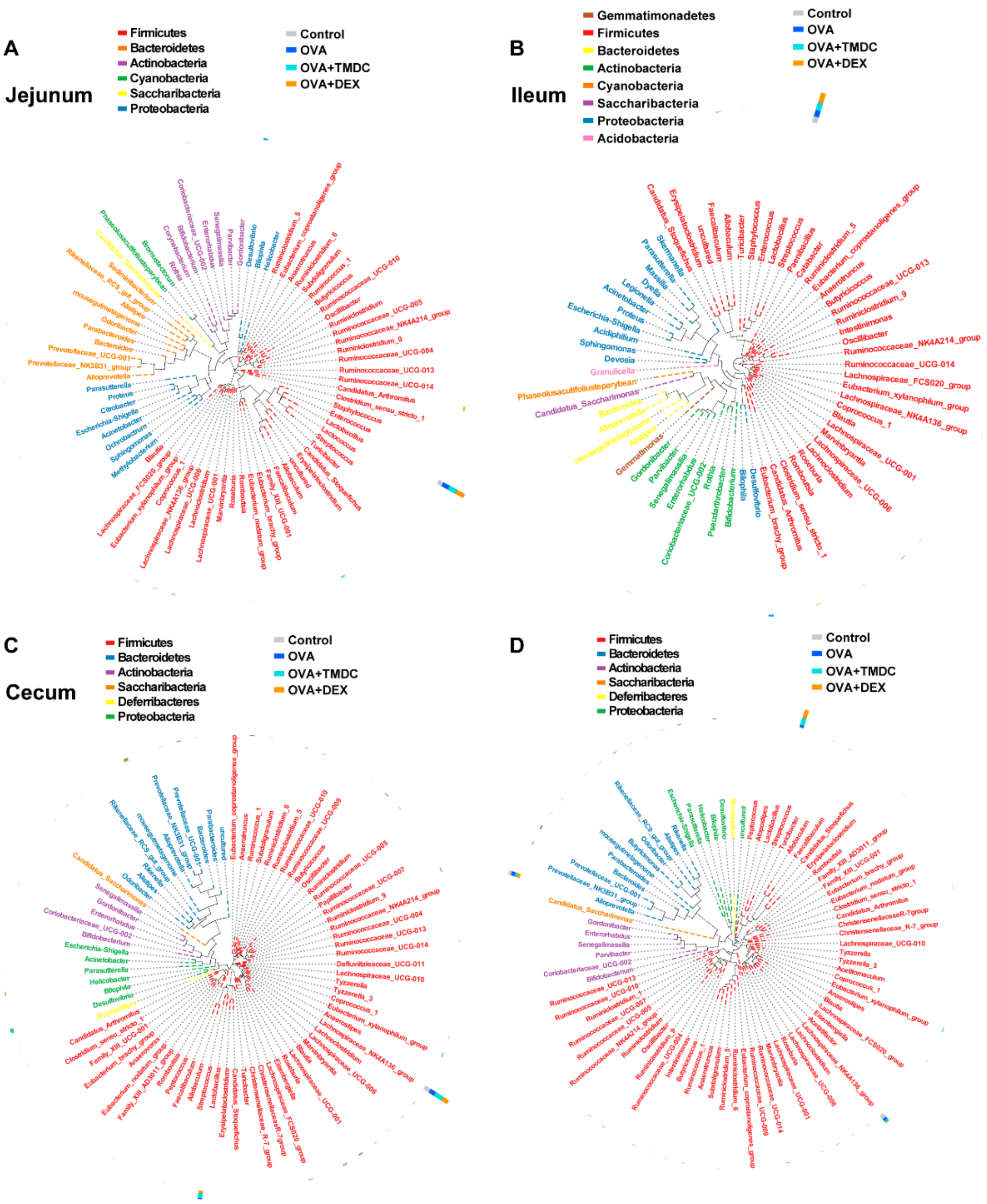

Supplementary Figure S1. Phylogenetic trees of gut microbiota across different intestinal segments. (A) Jejunum, (B) Ileum, (C) Cecum, (D) Colon.

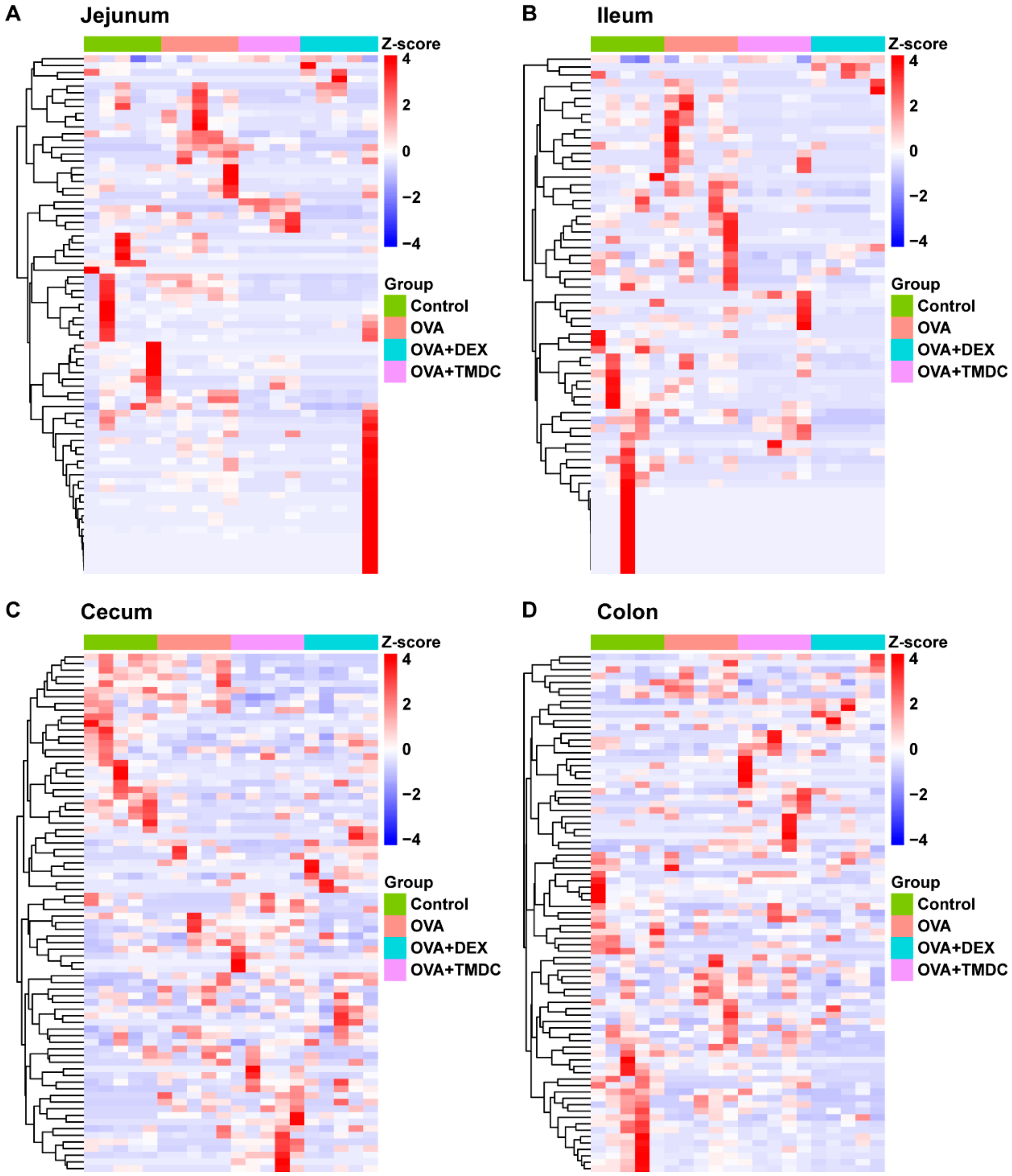

Supplementary Figure S2. Community clustering analyses of gut microbiota across different intestinal segments. (A) Jejunum, (B) Ileum, (C) Cecum, (D) Colon.

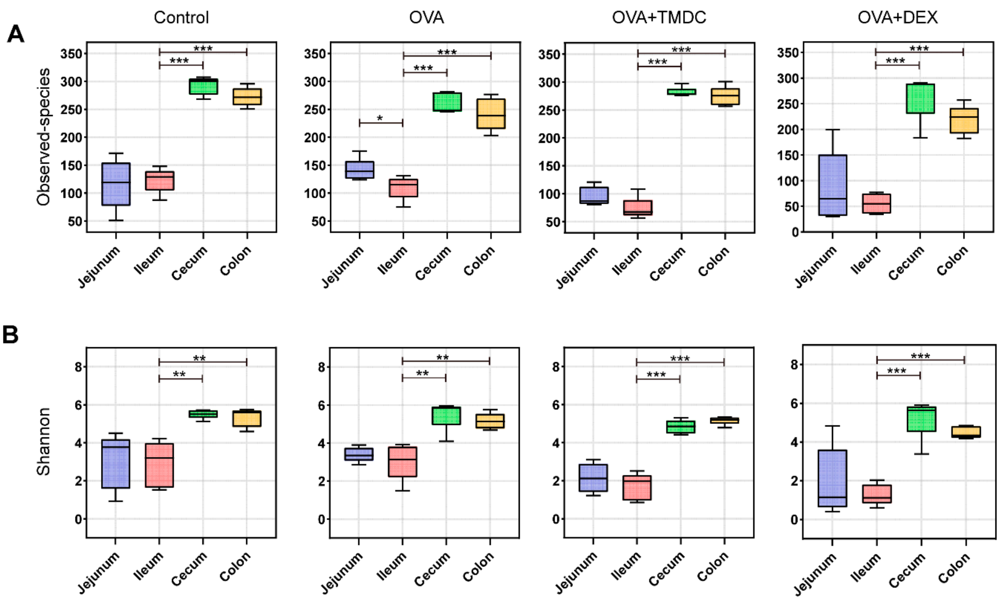

Supplementary Figure S3. The LI exhibited a higher alpha diversity than the SI of all mice**.** The alterations in the different intestinal microbiota structure of asthmatic mice. (A) Observed OTUs; (B) Shannon index. $^{**}P < 0.01$, $^{***}P < 0.001$.

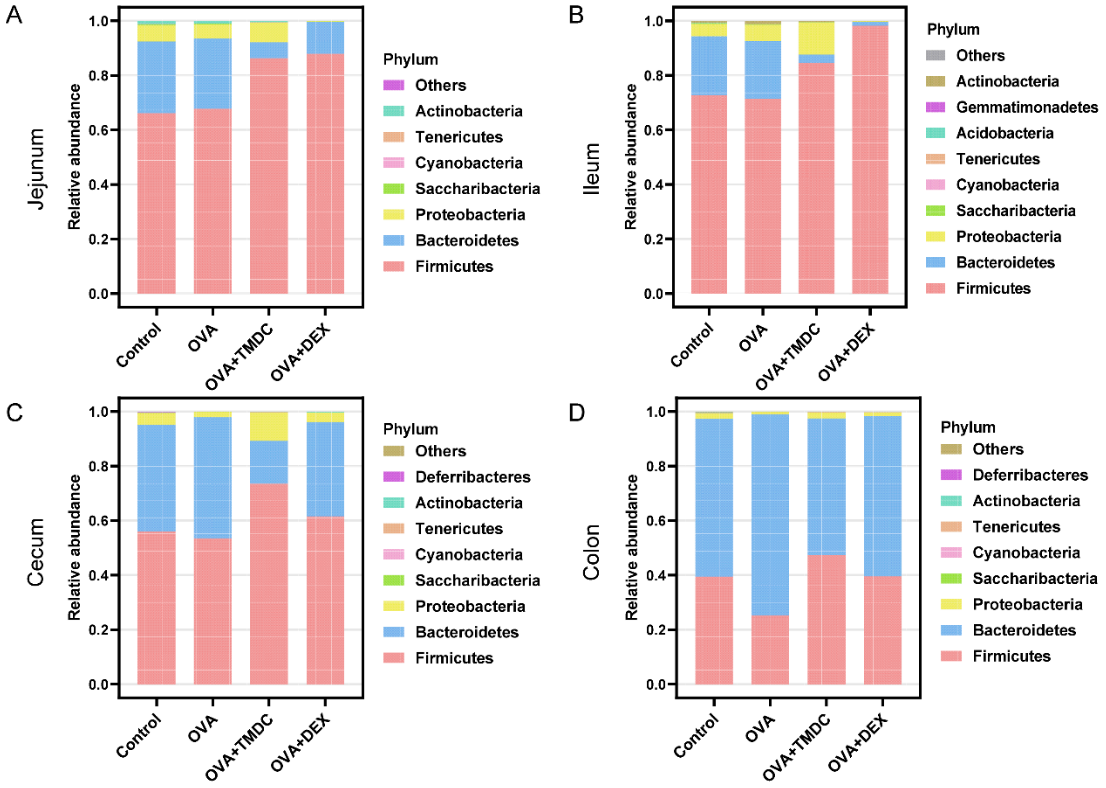

Supplementary Figure S4. Abundant phyla at different gut segments of mice.

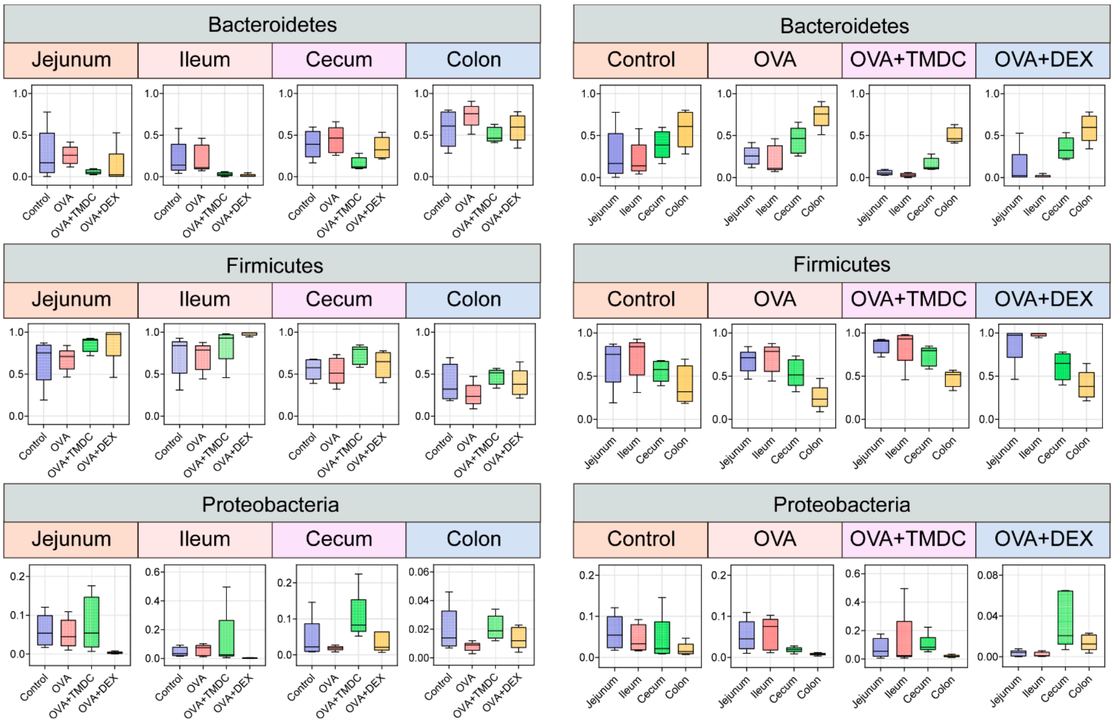

Supplementary Figure S5. Differentially abundant phyla at different gut segments among different groups.
